# Performance and microbial ecology of a nitritation sequencing batch reactor treating high-strength ammonia wastewater

**DOI:** 10.1038/srep35693

**Published:** 2016-10-20

**Authors:** Wenjing Chen, Xiaohu Dai, Dawen Cao, Sha Wang, Xiaona Hu, Wenru Liu, Dianhai Yang

**Affiliations:** 1 School of Environmental Science and Engineering, Tongji University, Shanghai 200092, P. R. China

## Abstract

The partial nitrification (PN) performance and the microbial community variations were evaluated in a sequencing batch reactor (SBR) for 172 days, with the stepwise elevation of ammonium concentration. Free ammonia (FA) and low dissolved oxygen inhibition of nitrite-oxidized bacteria (NOB) were used to achieve nitritation in the SBR. During the 172 days operation, the nitrogen loading rate of the SBR was finally raised to 3.6 kg N/m^3^/d corresponding the influent ammonium of 1500 mg/L, with the ammonium removal efficiency and nitrite accumulation rate were 94.12% and 83.54%, respectively, indicating that the syntrophic inhibition of FA and low dissolved oxygen contributed substantially to the stable nitrite accumulation. The results of the 16S rRNA high-throughput sequencing revealed that *Nitrospira*, the only nitrite-oxidizing bacteria in the system, were successively inhibited and eliminated, and the SBR reactor was dominated finally by *Nitrosomonas*, the ammonium-oxidizing bacteria, which had a relative abundance of 83%, indicating that the *Nitrosomonas* played the primary roles on the establishment and maintaining of nitritation. Followed by *Nitrosomonas, Anaerolineae* (7.02%) and *Saprospira* (1.86%) were the other mainly genera in the biomass.

There are many different activities that generate high-strength ammonium wastewaters including petrochemical effluent, pharmaceutical effluent, fertilizer waste and leachates produced by urban solid waste disposal sites. Some of the actual ammonium-rich wastewaters usually contain ammonium concentrations as high as 1000 mg/L^1^,^2^. Partial nitritation (PN) to nitrite has been reported to be technically feasible and economically favorable, especially when wastewater with high ammonium concentrations or low C/N ratios is treated^3^. However, under high ammonium concentrations, nitrogen overload might occur due to the sharp increase of influent ammonium concentration along with greater influent flow rate. The PN performance sometimes might be constrained with high free ammonia (FA). In addition, some problems have also been reported with the stability of the PN system because of the accumulation of nitrite-oxidizing bacteria (NOB) in the biomass under the nitrite-rich condition^4^.

To achieve PN, the activity of nitrite-oxidizing bacteria (NOB) must be selectively reduced without affecting the activity of ammonia-oxidizing bacteria (AOB)^5^. However, the low proliferation rates of AOB greatly limit the nitritation process. Thus, attempts have been made to develop new reactors or optimize operating conditions of the currently existing reactors. Several controlling factors can be applied to out-compete NOB and ensure nitrite accumulation, such as free ammonia (FA) or free nitrous acid (FNA) inhibition, dissolved oxygen (DO), pH, temperature and short sludge retention time (SRT)^6^,^7^. Among all factors, FA was commonly selected as a key parameter to achieve nitrite accumulation because of its different inhibition values on AOB and NOB^8^. Anthonisen *et al*.^9^ first reported that FA at 10–150 mg/L could inhibit *Nitrosomonas*, while 0.1–1.0 mg/L could inhibit *Nitrobcaters*^9^. Bae *et al*. reported that the influent FA concentrations were always in the inhibitory values (0.1–4.0 mg/L) for NOB^10^. From economic and practical considerations, DO concentration is a feasible control parameter, since low DO concentrations can save aeration consumption. In addition, the optimal pH for *Nitrosomonas spp.*, a typical AOB, ranges from 7.9 to 8.2, while that for *Nitrobacter spp.*, a typical NOB, ranges between 7.2 and 7.6, and that for PN reactor operation ranges between 7.6 and 8.2^11^,^12^. Moreover, it is believed that high temperature (30 °C–35 °C) is optimal for achieving nitritation^13^. All of these methods enhanced the start-up and stability of the nitritation reactor to a certain extent.

PN performance is crucially determined by the bacterial dynamics in the reactors, such as species and relative abundance of AOB. Thus, it is necessary to track the AOB dynamics during the startup and operation of nitritation reactors. Despite its significance, the AOB dynamics have not been fully revealed, possibly because of the limitations of conventional molecular methods such as the low sequencing depth^8^. The successful application of high-throughput sequencing technologies has made it possible to explore microbial community dynamics with sufficient sequencing depth^14^. Importantly, the high-throughput can make sequencing much more time- and cost-effective^15^,^16^ relative to conventional sequencing methods. Thus, it can facilitate comparative analysis among several samplings. It has been shown that high-throughput sequencing can provide significant information dealing with the population diversities of activated sludge^17^. However, few studies have investigated the microbial dynamics during the startup and operation of nitritation. Hopefully, high-throughput sequencing can improve our understanding of the nitritation process.

In this study, a sequencing batch reactor (SBR) was operated under influent ammonium concentrations up to 1500 mg/L to provide further information in the development of high-rate and stable operation of PN process treating high strength ammonium wastewater. The PN performance and the related stability of the nitrogen loading rate (NLR) were investigated during the 172 days operation. The DO and FA/FNA affecting PN performance were also evaluated accordingly. Variations in the microbial community populations during the SBR operation were examined by 16S rRNA high-throughput sequencing.

## Results and Discussion

### Reactor performance

**Figure 1:**
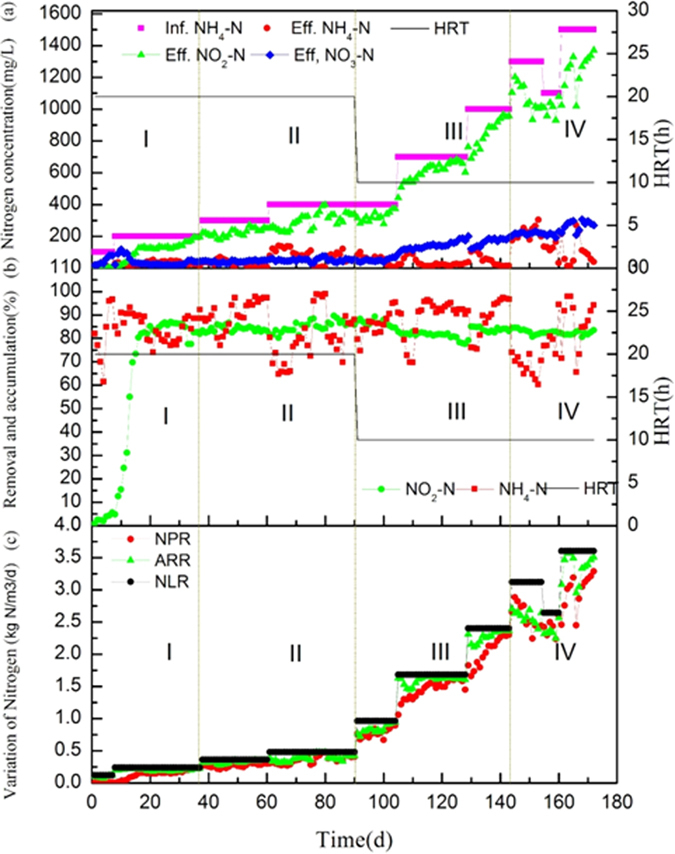
Performance of nitrogen removal in the NLR along the operational period. (**a**) Concentrations of nitrogen compounds and HRT. (**b**) Ammonium removal efficiency, NO_2_−N accumulation percentage and HRT. (**c**) NLR, ARR and NPR.

In this study, the SBR was operated for 172 days with the aim of obtaining stable PN and enriching AOB under high ammonium feeding. In the process, the influent ammonium concentration was increased progressively from 100 to 1500 mg/L, which led to the NLR gradually increasing. Based on the performance characteristics of the entire operation, the PN process could be divided into four phases (Fig. 1).

In phase I, during the first 7 days, the reactor was fed at low ammonium concentration (100 mg/L) and the ammonium nitrogen was primarily converted to nitrate rather than nitrite, suggesting that considerable levels of NOB were present in the reactor. As shown in Fig. 1, the operation was started with an initial NLR of 0.12 kg/m^3^/d. During the first 7 days of operation, a significant amount of ammonium was oxidized to nitrate (up to 80 mg/L on day 7, see Fig. 1a). As soon as the ammonium concentrations in the influent wastewater increased to 200 mg/L on day 8, nitrite accumulation occurred. When the NLR was increased from 0.12 to 0.24 kg/m^3^/d (Fig. 1c), the nitrate production progressively decreased to 28 mg/L (day 17), while the nitrite accumulation rate (NAR) increased from 4.85% (day 8) to 80% (day 17) (Fig. 1a,b). From day 17 onwards, stable partial nitritation was maintained in the reactor, and the average NAR was 82%, indicating a steady inhibition of NOB by the strategy of limited aeration and high free ammonia^18^.

In phase II, after the start-up period, the reactor was operated to elevate the NLR by increasing the influent ammonium concentration stepwise to test the PN performance at DO 0.8–1.0 mg/L. During this phase, the influent ammonium concentration was elevated to 300 mg/L, while the ammonium removal efficiency was maintained at above 85% at the fixed hydraulic retention time (HRT) of 20 h. During days 61–90, the influent ammonium was increased to 400 mg/L, the effluent nitrite progressively increased to 339 mg/L, and the system eventually reached the ammonium removal efficiency above 95% and a NAR of 85.5%. The ammonium removal rate (ARR) and nitrite production rate (NPR) were developed proportionately to 0.423 and 0.406 kg/m^3^/d respectively, as the NLR increased to 0.48 kg/m^3^/d.

A strong oxygen limiting condition was applied in the NLR by reducing the DO to 0.3–0.5 mg/L which was expected to enrich AOB^18^ and observe whether the decrease of DO concentration effect on the nitritation performance. In phase III (days 91–143), the influent ammonium was further raised to 1000 mg/L at a fixed HRT of 10 h, which corresponded to an NLR of 2.4 kg/m^3^/d, while the DO was decreased to 0.3–0.5 mg/L. The ARR was finally developed to 2.363 kg/m^3^/d, keeping the effluent ammonium at 12–24 mg/L. Although some ammonium fluctuations occurred in the effluent, the nitrification performance of the NLR was still maintained stable with average ammonium removal efficiency higher than 90%. Consequently, the effluent nitrite increased further to 958 mg/L and the NPR rose to 2.3 kg/m^3^/d with an NAR of 83% (average NAR around 82%).

Inhibited ammonium removal efficiency was observed in phase IV when the ammonium concentration increased to 1300 mg/L. As the experiment continued, the effluent ammonium increased to 266 mg/L, which corresponded to a gradual decrease in ammonium removal efficiency to 62%, but nitrite accumulation remained as high as 1018 mg/L. Then the ammonium level was decreased to 1100 mg/L, after 6 days of recovery, the ammonium removal efficiency gradually increased to 94.42%. Subsequently, the ammonium concentration increased to 1500 mg/L, which corresponded to the NLR was 3.6 kg N/m^3^/d, the ammonium removal efficiency and NAR finally reached to 94.12% and 83.54%, respectively. The NLR 3.6 kg N/m^3^/d was higher than many of the reported values (Table 1). These findings indicate that the system can be used to treat ammonium-rich wastewater with much higher ammonium concentrations at DO 0.3–0.5 mg/L.

### Mechanism of partial nitritation achievement in SBR

Oxygen limitation is a critical factor for maintaining PN stably via nitrite, and AOB outcompete NOB due to the stronger DO affinity of AOB than NOB at low DO concentrations^19^. It is known that a DO below 1.0 mg/L is a limiting factor, inhibiting the growth of NOB and alternatively enhancing the growth of AOB, which leads to nitrite accumulation^18^. Thus, the DO in the NLR was controlled at 0.8–1.0 mg/L in phase I and phase II to enrich AOB. As previously reported, the growth rate of AOB is 2.6 times faster than that of NOB at a DO level in the range of 0.5–1.0 mg/L^20^. With the restriction of DO in the range of 0.5–1.0 mg/L, nitrate production was significantly limited to a negligible amount and nitrite accumulated progressively.

Low level concentration of DO in the mixed liquor (0.3–0.5 mg/L) can help inhibiting the proliferation of NOB^19^. In phase III and phase IV, the DO was decreased to 0.3–0.5 mg/L, while the NLR was increased stepwise, finally reached to 3.6 kg/m^3^/d. Meanwhile, the effluent nitrite developed to 1370 mg/L and the NAR increased proportionately to higher than 80%. It should be noted that, when DO decreased from 0.8–1.0 mg/L (phase II) to 0.3–0.5 mg/L (phase III) with the constant influent ammonia concentration 400 mg/L, the average ammonia removal efficiency and the NAR have little fluctuated (Fig. 1b). These findings revealed that the PN process was insensitive to the DO concentration decreased from 0.8–1.0 mg/L to 0.3–0.5 mg/L with the constant influent ammonia concentration 400 mg/L.

FA and FNA are known to inhibit nitrification, especially nitrite oxidation^3^,^21^. Although many researchers have reported concentrations of FA and FNA that might inhibit the growth of NOB and cause the accumulation of AOB, the critical values recorded in these studies have varied^3^,^22^,^23^. NOB is inhibited by FA at concentrations ranging from 1 to 7 mg/L, while AOB begins to be inhibited at 150 mg/L^9^. Obviously, maintaining a relatively high FA concentration was a good strategy to suppress NOB and thus accumulate nitrite in the system. Additionally, researchers have reported that NOB was inhibited by FNA at 0.22 or 0.2 mg/L, while AOB was inhibited at 0.49 mg/L^9^,^13^.

**Figure 2:**
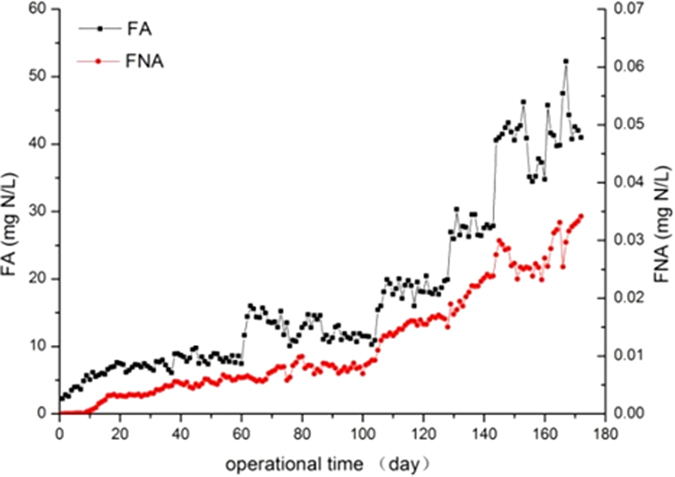
Concentrations of FA and FNA during nitrite accumulation. FA: start of cycle. FNA: end of cycle.

In this study, FA and FNA increased by increasing of ammonium concentration (Fig. 2). Moreover, we found that the concentration of FNA was below 0.2 mg/L, the valid inhibition value, throughout the experimental period; therefore, only the influence of FA on the performance was considered. The FA concentration of 2–4 mg/L in the influent played no role in the suppression of NOB. However, when the FA concentration rose above 5 mg/L, the nitrate production was dramatically inhibited, and nitrite build-up was noticed along the course of time. Later, in phase III, the FA was 10–30 mg/L, which was above the threshold inhibition values (10 mg/L) of AOB previously reported^24^. However, in our study, the ammonia removal efficiency was as high as 97% in phase III, revealing that the AOB was not suppressed under this FA concentration. Subsequently, in the following 10 days (Days 144–154) the influent FA further increased to 40–46 mg/L, which corresponded to an ammonium level of 1300 mg/L. Additionally, the much higher FA affected PN performance (as mentioned above), and the ammonia removal rate decreased from 82% to 62%. The influent ammonium concentration then decreased to 1100 mg/L, with an FA of 34–38 mg/L, after 6 days of recovery, at which time the ammonia removal rate increased to 94.42%. The ammonium concentration subsequently increased to 1500 mg/L, which corresponded to an FA of 40–52 mg/L, at which time the ammonia removal rate and NAR reached 94% and 84%, respectively. These findings suggest that the PN-SBR could stand a much higher FA concentration after a period of recovery.

### Microbial community analysis

Evaluation of the bacterial existing in the microbial community over the course of operation is helpful for considering the implementation of PN process treating high ammonium wastewater. To identify the microbial community dynamics during the SBR operation, high-throughput sequencing of bacterial 16S rRNA gene was performed using the Illumina Miseq platform. After removing the low quality reads, a total of 108,461 effective sequences were obtained for the eight samples. The sequence number for each sample was in the range of 9,086–18,162 (see Table 2), while the operational taxonomic units (OTUs) of each sample were in the range of 104–444 in the level of the 3% cutoff. Clearly, it can be seen that the species richness of the samples (except for S2) were obviously decreased during the long-term operation, as indicated by the smaller values of ACE and Chao 1. The species richness of the sample S2 collected at day 7 was obviously higher than that of the seed sludge (S1), while nitritation was not observed during the first 7 days (see Fig. 1). The species diversity of all samples was decreased, as indicated by the lower value of Shannon index during the whole operation. Finally, the OTUs number (104), chao 1 (160) and Shanon index (1.2) of S8 were significantly lower than that of the seed sludge sample (S1), revealing that the richness and diversity of microbial community in the nitritation system were significantly decreased under the long-term operation.

**Figure 3:**
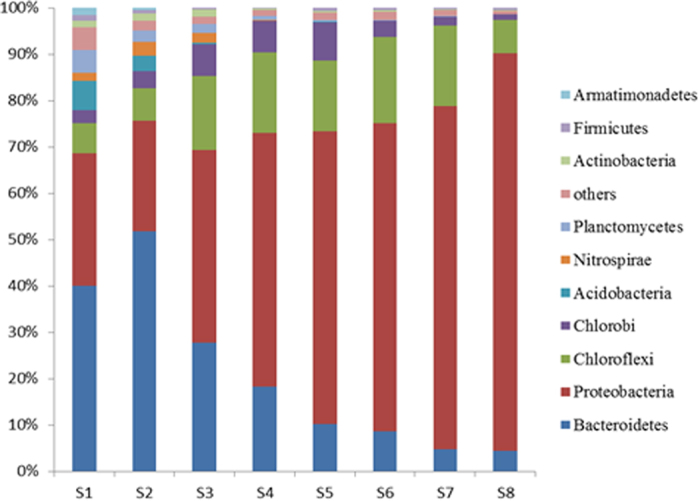
Relative abundance of major phyla in the eight samples. The eight samples were collected on days 0 (S1), 7 (S2), 34 (S3), 60 (S4), 90 (S5), 104 (S6), 142 (S7) and 172 (S8) respectively.

As shown in Fig. 3, *Proteobacteria* was the major phyla, with the exception of S2 (23.95%), the relative abundance of this phylum increased significantly over the entire operation (S8, 86.29%). Followed the *Proteobacteria, Chloroflexi* (7.2%), *Bacteroidetes* (4.4%) and *Chlorobi* (1.1%) were also the main dominant phyla in the end. In previous studies, denitrification and even chemolithotrophic denitrification by *Proteobacteria, Chlorobi* and *Chloroflexi* have been detected^25^,^26^,^27^,^28^. *Proteobacteria, Chloroflexi, Bacteroidetes* and *Chlorobi* were also the main dominant phyla in previous nitritation studies^29^,^30^. With the exception of S2 (52.11%), the relative abundance of *Bacteroidetes* decreased significantly from 38.57% (S1) to 4.41% (S8), which was less abundant than those reported studies regarding to nitritation process^29^,^30^. Interestingly, the relative abundance of the NOB *Nitrospirae* increased from 2% (S1) to 3% (S2) after 7 days. These findings indicate that NOB was an abundant phylum in S2, which was consistent with the low NAR in the SBR effluent on day 7 (see Fig. 1b), the nitrate concentrations in the effluent were high (>80 mg/L) on day 7. When the ammonia concentration increased to 200 mg/L after 25 days operation, the *Nitrospirae* decreased to 2% in sample of S3. *Nitrospirae* was undetected in samples of S5, S6, S7 and S8, implying that NOB disappeared from the reactor.

**Figure 4:**
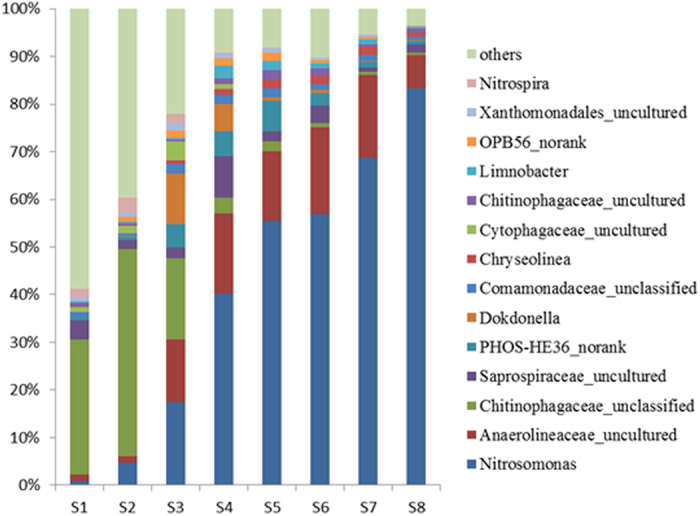
Top 14 genera in the eight samples. The eight samples were collected on days 0 (S1), 7 (S2), 34 (S3), 60 (S4), 90 (S5), 104 (S6), 142 (S7) and 172 (S8) respectively.

In order to further validate the function of the community, the top 14 genera determined according to their average abundance in the eight samples were illustrated in Fig. 4. *Nitrosomonas* were regarded as the dominant AOB and *Nitrospira* were the NOB in wastewater treatment plants (WWTPs)^31^. Obviously, *Nitrospira*, increased after 7 days from 1.85% (S1) to 3.07% (S2). These findings agree well with the *Nitrospirae* abundance at the phylum level in S1 and S2. As reported, the NOB *Nitrospira* are more sensitive to the high temperature and ammonium concentration than AOB *Nitrosomonas*^32^. Followed by the increased influent ammonia concentration, *Nitrospira* could not detected in the S5, S6, S7 and S8, probably confirmed the weak nitrite oxidation inside the reactor which was in accordance with nitritation performance. Specifically, the relative abundance of *Nitrosomonas* increased from 0.81% in S1 to 83% in S8, which was much higher than most of nitritation systems (10–69%)^33^. It suggested that the establishment and maintainance of nitritation was primarily the result of predominant AOB developed in the system. As shown in Fig. 4, slight variations occurred between S5 and S6, the relative abundance of *Nitrosomonas* reached 55.51% and 56.83% respectively, which indicated that there was little effect on the abundance of *Nitrosomonas* when DO concentration decreased from 0.8–1.0 mg/L to 0.3–0.5 mg/L with the constant influent ammonia concentration 400 mg/L. However, the *Nitrosomonas* genus was accompanied by a number of genera *Anaerolineae* (7.02%) and *Saprospira* (1.86%). Some species of *Anaerolineae* attached to the bacterial phylum *Chloroflexi* were known to degrade carbohydrate, which had a syntrophic relationship with hydrogenotrophic methanogens^34^. Members of *Saprospira* are generally associated with the degradation of complex organic materials^35^.

## Materials and Methods

### Materials

Synthetic wastewater containing ammonium chloride as a nitrogen source was used (see Table 3). Activated sludge was obtained from the secondary sedimentation tank of the Mudu wastewater treatment plant (WWTP) in Suzhou, Jiangsu Province, PR China. The WWTP receives wastewater from local industries (10%), including the pharmaceutical industry and the chemical industry.

### Partial nitrification reactor setup

The lab-scale SBR was constructed as a cylindrical tank with a total volume of 75 dm^3^ (40 cm in diameter and 60 cm high) and a working volume of 50 L. The reactor was equipped with a magnetic stirrer, as well as an air micro-diffuser at the bottom. The reactor was operated at a prolonged sludge residence time (SRT), with no sludge discharged during the experiment, except for the sludge taken during sampling.

Initially, the SBR was inoculated with activated sludge and fed synthetic wastewater using a peristaltic pump. The final suspended solids (SS) and volatile suspended solids (VSS) levels of the inoculation in the reactor were 4.6 and 2.1 g/L, respectively, which corresponded to a VSS/SS of 0.5. In the experiment, DO, pH and T were automatically controlled by a programmable logic controller. The DO concentration in the bulk liquid was measured on-line by a DO electrode (WTW Oxi 340i CellOx 325), and automatically controlled at 0.8–1.0 mg/L in phase I and phase II, then controlled at 0.3–0.5 mg/L in phase III and phase IV to enrich AOB in the system, because AOB have higher tolerance for O_2_ and NO_2_^−^ and higher growth rates at high ammonia concentrations^36^. The pH was measured online with a pH probe (WTW SenTix 950) and automatically controlled at 7.8–8.0 by dosing with 0.5 M Na_2_CO_3_. Temperature was controlled at 30.0 ± 0.1 using an electrical heating device. The SBR operating conditions are shown in detail in Table 4. The SBR cycle consisted of four phases: 15-min feeding, 9-h aeration, 30-min settling, and 15-min decanting in phase I and phase II, while 15-min feeding, 4-h aeration, 30-min settling, and 15-min decanting in phase III and phase IV. The discharge ratio was 50%.

### Wastewater quality analysis

Analytical measurements of ammonium (NH_3_-N), nitrite (NO_2_-N), nitrate (NO_3_-N), SS and VSS in the system were performed according to the Standard Methods^37^.

### High-throughput 16S rRNA gene pyrosequencing and data analysis

Eight samples for Illumina high-throughput sequencing were obtained from different periods of the operation (S1–S8). Total DNA of the samples was extracted using a Soil DNA Kit (OMEGA, USA) according to the manufacturer’s instructions. DNA samples were amplified in triplicate by PCR using primer set F515 (5′-GTGCCAGCMGCCGCGG-3′) and R907 (5′-CCGTCAATTCMTTTRAGTTT-3′) for the V4-V5 region of the 16S rRNA gene. The 12-nucleotide barcodes were added to the 5′ end of R907 to allow multiplexing. Details regarding the PCR procedures can be found elsewhere^38^. The quality of PCR products was assessed using agarose gel electrophoresis, after which they were purified with AxyPrep DNA gel recovery kits (AXYGEN, USA) and quantified using a Nanodrop spectrophotometer. Next, 16S rRNA gene sequencing was conducted using the Illumina Miseq platform based on paired-end sequencing. Acquired Illumina reads with low-quality, such as those with a length shorter than 150 bp or containing one or more ambiguous bases, were removed from the sequencing datasets using RDP tools (http://pyro.cme.msu.edu/). In addition, the alpha diversity (Chao 1, ACE, Simpson and Shannon) of the sequences was calculated following the pipeline of QIIME^39^. Taxonomic classifications of the quality-controlled sequences were then conducted using RDP Classifier with a confidence threshold of 80%. Operational taxonomic units (OTU) were acquired from the sequences using UCLUST at a 97% similarity level^40^.

### Calculations

The nitrite accumulation ratio (NAR, %) was calculated using Eq. (1).


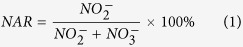


The free ammonia (FA) and free nitrous acid (FNA) concentrations were calculated using the acid-base equilibria (Eqs (2) and (3))^41^.


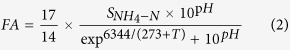



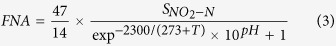


## Additional Information

**How to cite this article**: Chen, W. *et al*. Performance and microbial ecology of a nitritation sequencing batch reactor treating high-strength ammonia wastewater. *Sci. Rep.* **6**, 35693; doi: 10.1038/srep35693 (2016).

## Figures and Tables

**Table 1:** Comparison of the SBR performances with several reference works under high ammonium condition.

system	volume (L)	DO (mg/L)	NH^4^−N (mg/L)	NAR (%)	NLR (kg/m^3^/d)	ARR (kg/m^3^/d)	references
Activated sludge unit	21.0	<2.0	3300	16–20	1.0–4.0		1
Swim-bed reactor	10.8	8.01 min^−1^	1000	95.4	5.45	—	42
Activated sludge system	26.0	6.5	1350	98	1.2	—	43
Internal loop airlift reactor	3.8	1.1–2.1	1039	—	—	—	2
Airlift reactor	5.0	2.2	1400	≥80	2.1	2.0	23
SBR	50	0.3–0.5	1500	83.5	3.6	3.5	This study

**Table 2:** Diversity and richness of the 16S rRNA sequences.

Samples	Reads	OTUs	ACE^a^	Chao^a^	Shannon^b^	Simpson^c^
S1	9,086	444	482	480	4.3	0.08
S2	10,952	431	506	502	3.5	0.19
S3	18,162	404	483	497	3.7	0.07
S4	12,121	181	221	228	3.1	0.11
S5	12,658	163	189	191	2.7	0.20
S6	12,802	125	152	150	2.2	0.29
S7	17,871	120	140	132	1.7	0.44
S8	14,809	104	142	160	1.2	0.62

^a^Community richness. A higher number indicates more richness.

^b^Community diversity. A higher number indicates more diversity.

^c^Community diversity. A higher number indicates less diversity.

**Table 3:** Components of the synthetic wastewater.

Components	Concentration (g/L)	Components	Concentration (g/L)
NH_4_Cl	Add as needed	CaCl_2_ · H_2_O	0.14
KH_2_PO_4_	0.025	MgSO_4_ · 7H_2_O	0.3
Trace elements 5 ml/L			
CoSO_4_ · H_2_O	1.9	ZnSO_4_ · 7H_2_O	5
MnCl_2_ · 4H_2_O	5.06	CuSO_4_ · 5H_2_O	1.57
Na_2_-EDTA	50	FeSO_4_ · 7H_2_O	5
NaMoO_4_ · 2H_2_O	1.1		

**Table 4:** The operation condition in different period.

Operational period	Time (days)	HRT (h)	nitrogen loading rate (kg N/m^3^/d)	DO (mg/L)
I	1–37	20	0.12–0.24	0.8–1.0
II	38–90	20	0.36–0.48	0.8–1.0
III	91–143	10	0.96–2.4	0.3–0.5
IV	144–172	10	3.12–3.6	0.3–0.5

## References

[b1] Campos J. L., Mosquera-Corral A., Sanchez M., Mendez R. & Lema J. M. Nitrification in saline wastewater with high ammonia concentration in an activated sludge unit. Water Res 36, 2555–2560 (2002). 12153022 10.1016/s0043-1354(01)00467-5

[b2] Jin R. C., Xing B. S. & Ni W. M. Optimization of partial nitritation in a continuous flow internal loop airlift reactor. Bioresource Technol 147, 516–524 (2013). 10.1016/j.biortech.2013.08.077 24012847

[b3] Peng Y. Z. & Zhu G. B. Biological nitrogen removal with nitrification and denitrification via nitrite pathway. Appl Microbiol Biot 73, 15–26 (2006). 10.1007/s00253-006-0534-z 17028876

[b4] Fux C., Huang D., Monti A. & Siegrist H. Difficulties in maintaining long-term partial nitritation of ammonium-rich sludge digester liquids in a moving-bed biofilm reactor (MBBR). Water Sci Technol 49, 53–60 (2004). 15303723

[b5] Fudala-Ksiazek S., Luczkiewicz A., Fitobor K. & Olanczuk-Neyman K. Nitrogen removal via the nitrite pathway during wastewater co-treatment with ammonia-rich landfill leachates in a sequencing batch reactor. Environ Sci Pollut R 21, 7307–7318 (2014). 10.1007/s11356-014-2641-1 PMC4053604 24569868

[b6] Ahn J. H., Kwan T. & Chandran K. Comparison of partial and full nitrification processes applied for treating high-strength nitrogen wastewaters: microbial ecology through nitrous oxide production. Environ Sci Technol 45, 2734–2740 (2011). 21388173 10.1021/es103534g

[b7] Liang Z. & Liu J. X. Control factors of partial nitritation for landfill leachate treatment. Journal of environmental sciences 19, 523–529 (2007). 10.1016/s1001-0742(07)60087-4 17915679

[b8] Zhang T., Shao M. F. & Ye L. 454 Pyrosequencing reveals bacterial diversity of activated sludge from 14 sewage treatment plants. Isme J 6, 1137–1147 (2012). 22170428 10.1038/ismej.2011.188 PMC3358032

[b9] Anthonisen A. C., Loehr R. C., Prakasam T. B. & Srinath E. G. Inhibition of nitrification by ammonia and nitrous acid. Journal - Water Pollution Control Federation 48, 835–852 (1976). 948105

[b10] Bae W., Baek S., Chung J. & Lee Y. Optimal operational factors for nitrite accumulation in batch reactors. Biodegradation 12, 359–366 (2001). 11995828 10.1023/a:1014308229656

[b11] Shalini S. S. & Joseph K. Nitrogen management in landfill leachate: Application of SHARON, ANAMMOX and combined SHARON-ANAMMOX process. Waste Manage 32, 2385–2400 (2012). 10.1016/j.wasman.2012.06.006 22766438

[b12] Daalkhaijav U. & Nemati M. Ammonia loading rate: an effective variable to control partial nitrification and generate the anaerobic ammonium oxidation influent. Environ Technol 34, 2907–U2980 (2013). 10.1080/09593330.2013.796006 24645430

[b13] Hellinga C., Schellen A. A. J. C., Mulder J. W., van Loosdrecht M. C. M. & Heijnen J. J. The SHARON process: An innovative method for nitrogen removal from ammonium-rich waste water. Water Sci Technol 37, 135–142 (1998).

[b14] Shendure J. & Ji H. L. Next-generation DNA sequencing. Nat Biotechnol 26, 1135–1145 (2008). 18846087 10.1038/nbt1486

[b15] Loman N. J. . Performance comparison of benchtop high-throughput sequencing platforms (vol 30, pg 434, 2012). Nat Biotechnol 30, 562–562 (2012). 10.1038/nbt.2198 22522955

[b16] Zhang H. S. Using pyrosequencing and quantitative PCR to analyze microbial communities (vol 5, pg 21, 2011). Front Environ Sci En 5, 488–488 (2011).

[b17] Ma J. X., Wang Z. W., Yang Y., Mei X. J. & Wu Z. C. Correlating microbial community structure and composition with aeration intensity in submerged membrane bioreactors by 454 high-throughput pyrosequencing. Water Res 47, 859–869 (2013). 23200801 10.1016/j.watres.2012.11.013

[b18] Chuang H. P., Ohashi A., Imachi H., Tandukar M. & Harada H. Effective partial nitrification to nitrite by down-flow hanging sponge reactor under limited oxygen condition. Water Res 41, 295–302 (2007). 17141821 10.1016/j.watres.2006.10.019

[b19] Guo J. . Long-term effect of dissolved oxygen on partial nitrification performance and microbial community structure. Bioresource Technol 100, 2796–2802 (2009). 10.1016/j.biortech.2008.12.036 19201600

[b20] Park H. D. & Noguera D. R. Evaluating the effect of dissolved oxygen on ammonia-oxidizing bacterial communities in activated sludge. Water Res 38, 3275–3286 (2004). 15276744 10.1016/j.watres.2004.04.047

[b21] Ma J. . Effect of free nitrous acid as inhibitors on nitrate reduction by a biological nutrient removal sludge. J Hazard Mater 175, 518–523 (2010). 19910113 10.1016/j.jhazmat.2009.10.036

[b22] Kim J. H., Guo X. J. & Park H. S. Comparison study of the effects of temperature and free ammonia concentration on nitrification and nitrite accumulation. Process Biochem 43, 154–160 (2008).

[b23] Chai L. Y. . Partial nitrification in an air-lift reactor with long-term feeding of increasing ammonium concentrations. Bioresour Technol 185, 134–142 (2015). 25768415 10.1016/j.biortech.2015.02.091

[b24] Mosquera-Corral A., Gonzalez F., Campos J. L. & Mendez R. Partial nitrification in a SHARON reactor in the presence of salts and organic carbon compounds. Process Biochem 40, 3109–3118 (2005).

[b25] Fernandez N., Sierra-Alvarez R., Field J. A., Amils R. & Sanz J. L. Microbial community dynamics in a chemolithotrophic denitrification reactor inoculated with methanogenic granular sludge. Chemosphere 70, 462–474 (2008). 17689587 10.1016/j.chemosphere.2007.06.062

[b26] Koenig A., Zhang T., Liu L. H. & Fang H. H. Microbial community and biochemistry process in autosulfurotrophic denitrifying biofilm. Chemosphere 58, 1041–1047 (2005). 15664612 10.1016/j.chemosphere.2004.09.040

[b27] Mao Y., Xia Y. & Zhang T. Characterization of Thauera-dominated hydrogen-oxidizing autotrophic denitrifying microbial communities by using high-throughput sequencing. Bioresour Technol 128, 703–710 (2013). 23247099 10.1016/j.biortech.2012.10.106

[b28] Zhang L., Zhang C., Hu C., Liu H. & Qu J. Denitrification of groundwater using a sulfur-oxidizing autotrophic denitrifying anaerobic fluidized-bed MBR: performance and bacterial community structure. Appl Microbiol Biotechnol 99, 2815–2827 (2015). 25343972 10.1007/s00253-014-6113-9

[b29] Wang B., Peng Y., Guo Y., Zhao M. & Wang S. Illumina MiSeq sequencing reveals the key microorganisms involved in partial nitritation followed by simultaneous sludge fermentation, denitrification and anammox process. Bioresour Technol 207, 118–125 (2016). 26874440 10.1016/j.biortech.2016.01.072

[b30] Dosta J. . Two-step partial nitritation/Anammox process in granulation reactors: Start-up operation and microbial characterization. Journal of environmental management 164, 196–205 (2015). 26386756 10.1016/j.jenvman.2015.08.023

[b31] Ye L. & Zhang T. Estimation of nitrifier abundances in a partial nitrification reactor treating ammonium-rich saline wastewater using DGGE, T-RFLP and mathematical modeling. Appl Microbiol Biotechnol 88, 1403–1412 (2010). 20737268 10.1007/s00253-010-2837-3

[b32] Gonzalez-Martinez A., Poyatos J. M., Hontoria E., Gonzalez-Lopez J. & Osorio F. Treatment of effluents polluted by nitrogen with new biological technologies based on autotrophic nitrification-denitrification processes. Recent patents on biotechnology 5, 74–84 (2011). 21619549 10.2174/187220811796365671

[b33] Tokutomi T., Shibayama C., Soda S. & Ike M. A novel control method for nitritation: The domination of ammonia-oxidizing bacteria by high concentrations of inorganic carbon in an airlift-fluidized bed reactor. Water Res 44, 4195–4203 (2010). 20554306 10.1016/j.watres.2010.05.021

[b34] Narihiro T. . Quantitative detection of previously characterized syntrophic bacteria in anaerobic wastewater treatment systems by sequence-specific rRNA cleavage method. Water Res 46, 2167–2175 (2012). 22342314 10.1016/j.watres.2012.01.034

[b35] Xia Y., Kong Y., Thomsen T. R. & Halkjaer Nielsen P. Identification and ecophysiological characterization of epiphytic protein-hydrolyzing saprospiraceae (“Candidatus Epiflobacter” spp.) in activated sludge. Applied and environmental microbiology 74, 2229–2238 (2008). 18263744 10.1128/AEM.02502-07 PMC2292613

[b36] Schramm A., De Beer D., Gieseke A. & Amann R. Microenvironments and distribution of nitrifying bacteria in a membrane-bound biofilm. Environmental microbiology 2, 680–686 (2000). 11214800 10.1046/j.1462-2920.2000.00150.x

[b37] APHA. Standard Methods for the Examination of Water and Wastewater. American Public Health Association, Washington, DC. 47, 859–869 (1998).

[b38] Meng F. G. . A novel nonwoven hybrid bioreactor (NWHBR) for enhancing simultaneous nitrification and denitrification. Biotechnol Bioeng 110, 1903–1912 (2013). 23436222 10.1002/bit.24866

[b39] Caporaso J. G. . QIIME allows analysis of high-throughput community sequencing data. Nat Methods 7, 335–336 (2010). 20383131 10.1038/nmeth.f.303 PMC3156573

[b40] Edgar R. C. Search and clustering orders of magnitude faster than BLAST. Bioinformatics 26, 2460–2461 (2010). 20709691 10.1093/bioinformatics/btq461

[b41] Ford D. L., Churchwell R. L. & Kachtick J. W. Comprehensive analysis of nitrification of chemical processing wastewaters. J. Water Pollut. Con. Fed.S2 73, 2726–2746 (1980).

[b42] Qiao S. . High-rate partial nitrification performance of high ammonium containing wastewater under low temperatures. Bioresour Technol 101, 111–117 (2010). 19709879 10.1016/j.biortech.2009.08.003

[b43] Tora J. A., Lafuente J., Baeza J. A. & Carrera J. Long-term starvation and subsequent reactivation of a high-rate partial nitrification activated sludge pilot plant. Bioresour Technol 102, 9870–9875 (2011). 21890345 10.1016/j.biortech.2011.08.008

